# Combination Effect of Cilengitide with Erlotinib on TGF-β1-Induced Epithelial-to-Mesenchymal Transition in Human Non-Small Cell Lung Cancer Cells

**DOI:** 10.3390/ijms23073423

**Published:** 2022-03-22

**Authors:** Jisu Jeong, Jiyeon Kim

**Affiliations:** Department of Medical Laboratory Science, School of Health Science, Dankook University, Cheonan 31116, Korea; jjs0892@hanmail.net

**Keywords:** NSCLC, A549, erlotinib, cilengitide, TGF-β1, EMT

## Abstract

The epithelial-to-mesenchymal transition (EMT) is important for morphogenesis during development and is mainly induced by transforming growth factor (TGF)-β. In lung cancer, EMT is characterized by the transformation of cancer cells into a mobile, invasive form that can transit to other organs. Here, using a non–small cell lung cancer (NSCLC) cell line, we evaluated the EMT-related effects of the epidermal growth factor receptor inhibitor erlotinib alone and in combination with cilengitide, a cyclic RGD-based integrin antagonist. Erlotinib showed anti-proliferative and inhibitory effects against the TGF-β1–induced EMT phenotype in NSCLC cells. Compared with erlotinib alone, combination treatment with cilengitide led to an enhanced inhibitory effect on TGF-β1–induced expression of mesenchymal markers and invasion in non–small cell lung cancer A549 cells. These results suggest that cilengitide could improve anticancer drug efficacy and contribute to improved treatment strategies to inhibit and prevent EMT-based cancer progression.

## 1. Introduction

Among cancer deaths, lung cancer accounts for the largest share, with an incidence rate that is increasing every year worldwide [1]. Lung cancer occurs as one of two broad types, small cell lung cancer and non–small cell lung cancer (NSCLC). Of these, NSCLC accounts for approximately 80–85% of cases [1]. The NSCLC also has been classified into three main types: adenocarcinoma, squamous cell carcinoma, and large cell [2]. Depending on the epithelial nature of lung cancer, adenocarcinomas (approximately 70–75% of NSCLC) exhibit a completely different epithelial phenotype (derived from simple epithelium), whereas squamous cell carcinomas or epidermoid carcinomas of lung (25–30%) cancer are derived from keratinocytes or squamous (multilayered) epithelia of the lung. In these two tumor subtypes, cellular plasticity-related processes such as EMT and invasiveness have different properties and require different molecular mechanisms [2]. The process of cell conversion from epithelial to mesenchymal, known as the epithelial-to-mesenchymal transition (EMT), is normal in embryonic development, wound healing, and fibrosis [1,3]. In NSCLC, however, this transition represents a cellular shift from polarized, tightly bound epithelial cells to the motile and invasive fusiform mesenchymal cells of metastasis. For this reason, a candidate strategy for preventing NSCLC metastasis and invasion to other organs is inhibiting pathways associated with EMT, in addition to the use of conventional therapeutic drugs.

Among the factors that trigger the EMT process, one of the most important is a cytokine, transforming growth factor (TGF)-β [4,5,6,7]. In TGF-β–induced EMT, TGF-β binds to the transmembrane Ser/Thr receptors TGF-β type I (TβR-I) and type II (TβR-II), triggering phosphorylation of TβR-I, followed by downstream phosphorylation of the effectors Smad2 and Smad3 [8,9]. The phosphorylated Smad2/3 recruits Smad4 to form a complex, then translocates from the cytoplasm to the nucleus and binds to transcription factors such as Snail/Slug and Twist to activate EMT-related genes [10,11,12]. The EMT process also can occur through non-Smad signaling pathways such as Wnt/β-catenin or MAPK/ERK [13,14,15].

Erlotinib (trade name Tarceva) is an epidermal growth factor receptor (EGFR) tyrosine kinase inhibitor (TKI) in NSCLC cells and, along with gefitinib, is a first-generation TKI used to treat NSCLC with EGFR mutation [16]. Erlotinib is approved by the U.S. Food and Drug Administration as a second-line treatment for NSCLC that has not responded to first-line treatment. Because long-term use can lead to resistance and side effects, it is combined with other therapies [17,18]. Various treatment strategies for overcoming the side effects of chemotherapy for NSCLC are being studied [19,20,21,22,23,24,25].

Cilengitide is a cyclic peptide based on a tripeptide RGD (arginine, glycine, aspartic acid)-containing structure and is used to increase therapeutic efficacy along with various drugs for the treatment of several tumors [26]. Since integrins involved in angiogenesis and metastasis in the tumor microenvironment can be recognized by substances containing RGD sequences, RGD-containing peptides or peptidomimetics targeting integrins can be utilized as candidates for anticancer chemotherapy [27,28,29]. Several in vitro or clinical studies have demonstrated the efficacy of cilengitide and the effect of co-administration with existing anticancer drugs, but few studies have been conducted on the mechanism for EMT inhibition in NSCLC and its combination with cancer therapeutics [30,31,32,33,34,35]. In addition, we recently described an enhancing effect of cilengitide with gefitinib on limiting TGF-β–mediated EMT in NSCLC cells. By inhibiting Smad and non-Smad signaling, cilengitide enhanced the effect of gefitinib on NSCLC cell death and TGF-β–induced EMT phenotype change without severe cytotoxicity in normal cells [36].

Here, we investigated the anticancer effect of erlotinib and its inhibition of TGF-β–induced EMT. We also assessed whether the combination of erlotinib and cilengitide could enhance the inhibitory effects of erlotinib on TGF-β–induced EMT while minimizing side effects.

## 2. Results

### 2.1. Erlotinib Inhibits Tgf-Β1–Induced Emt-Related Phenotype Changes

**Figure 1:**
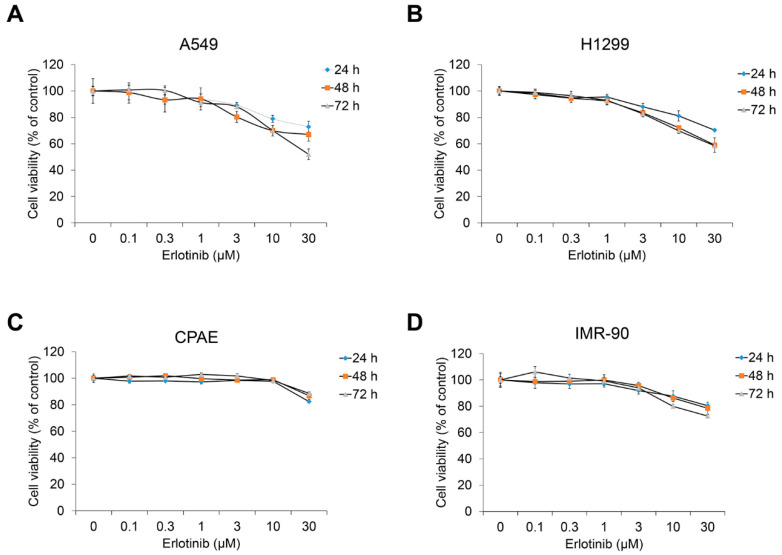
Erlotinib inhibits proliferation of human NSCLC A549 and H1299 cells. (**A**) A549, (**B**) H1299, (**C**) CPAE, and (**D**) IMR90 cells were treated with erlotinib for 24–72 h. After incubation, cell viability was measured by the EZ-Cytox Enhanced Cell Viability Assay Kit. Experiments were performed in triplicate. Data represent mean ± SD.

Before evaluating the effect of erlotinib on the TGF-β1–induced EMT process, we investigated cell viability in NSCLC A549, H1299, normal lung epithelium CPAE, and normal lung fibroblast IMR90 cells. Treatment with an erlotinib concentration of 1 μm or higher reduced the viability of A549 and H1299 cells (Figure 1A,B) but scarcely reduced the viability of CPAE cells (Figure 1C). In addition, the viability of IMR90 was slightly decreased compared with A549 and H1299.

**Figure 2:**
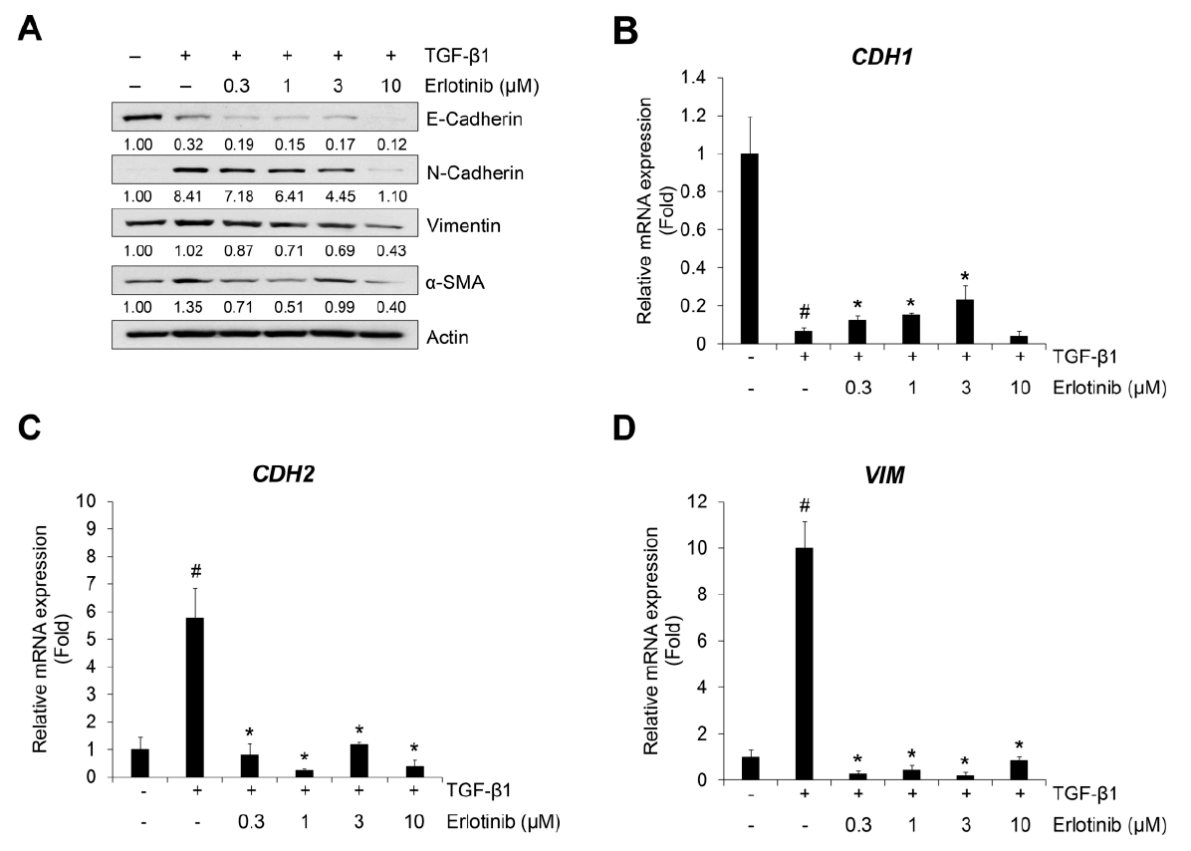
Erlotinib inhibits TGF-β1–induced expression of EMT markers. (**A**) Serum-deprived A549 cells were treated with TGF-β1 (5 ng/mL) or in combination with erlotinib for 72 h. Protein expression of an epithelial marker (E-cadherin) and mesenchymal markers (N-cadherin, vimentin, and α-smooth muscle actin) was determined by Western blot analysis. Actin was used as a loading control. (**B**–**D**) Serum-deprived A549 cells were incubated with TGF-β1 (5 ng/mL) and erlotinib for 48 h. After RNA extraction and cDNA synthesis, we performed qRT-PCR to measure the expression of *CDH1*, *CDH2*, and *VIM* mRNA using GAPDH as an internal control. ^#^ *p* < 0.01 versus control; * *p* < 0.05 versus the group treated with TGF-β1 only.

TGF-β1 reduces expression of the epithelial marker E-cadherin but induces an increase in the mesenchymal markers N-cadherin, vimentin, and α-SMA [37,38]. For this reason, we checked the effect of erlotinib on the TGF-β1–induced EMT-related phenotype changes in NSCLC A549 and H1299 cells. As shown in Figure 2A and Figure S4, erlotinib suppressed expression of TGF-β1–related increases in N-cadherin, vimentin, and α-SMA in A549 and H1299 cells. However, the EMT-related decreased expression of E-cadherin did not fully recover with erlotinib. Moreover, in CPAE cells, erlotinib did not suppress the expression of EMT-related genes according to the concentration gradient (Figure S5).

The inhibitory effects of erlotinib were confirmed using the TGF-β1–mediated transcription of epithelial and mesenchymal marker genes *CDH1*, *CDH2*, and *VIM* (encoding E-cadherin, N-cadherin, and vimentin, respectively). Treatment with TGF-β1 decreased expression of *CDH1* mRNA (Figure 2B) and increased expression of *CDH2* (Figure 2C) and *VIM* mRNA (Figure 2D). However, the expression of *CDH1* mRNA slightly recovered at 1 μM, and the expression of *CDH2* and *VIM* mRNA was inhibited at erlotinib concentrations >1 μM. These results indicate that erlotinib concentrations >1 μM have inhibitory effects on TGF-β1–induced EMT-related phenotypic changes and related gene expression in A549 cells.

### 2.2. Erlotinib Inhibits Tgf-Β1–Induced Smad and Non-Smad Signaling

**Figure 3:**
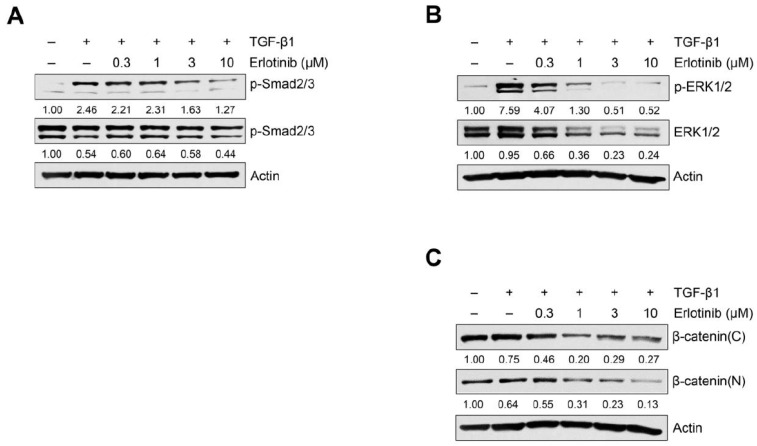
Erlotinib inhibits TGF-β1–induced Smad and non-Smad signaling pathways. Serum-deprived A549 cells were treated with TGF-β1 (5 ng/mL) with erlotinib for 48 h (**A**) or 72 h (**B**,**C**). Protein expression of the p-Smad2/3 (**A**), p-ERK1/2 (**B**), β-catenin (**C**), and endogenous forms of Smad2/3 and ERK1/2 was determined by Western blot analysis. Actin was used as a loading control.

Because the TGF-β1–induced EMT process could be modulated through the activation of Smad or non-Smad signaling [8,9,13,14,15], we investigated the effect of erlotinib on TGF-β1–induced Smad and non-Smad signaling pathways in A549 and H1299 cells. As shown in Figure 3A, erlotinib suppressed phosphorylation of Smad2/3. To determine whether erlotinib inhibits non-Smad signaling, we measured EGFR-triggered phosphorylation of downstream MAPK ERK1/2 and translocation of β-catenin into the nucleus. As shown in Figure 3B, Figures S6 and S7, erlotinib inhibited TGF-β1–induced phosphorylation of ERK1/2 and nuclear localization of β-catenin. In particular, erlotinib inhibited phosphorylation of the downstream target ERK1/2 more strongly than it inhibited Smad2/3 phosphorylation. These results suggest that erlotinib has an inhibitory effect on both Smad and non-Smad signaling mediating EMT progression in NSCLC cells.

### 2.3. Combination Cilengitide with Erlotinib Exerts a Synergistic Inhibitory Effect on NSCLC Cell Viability and EMT Markers

**Figure 4:**
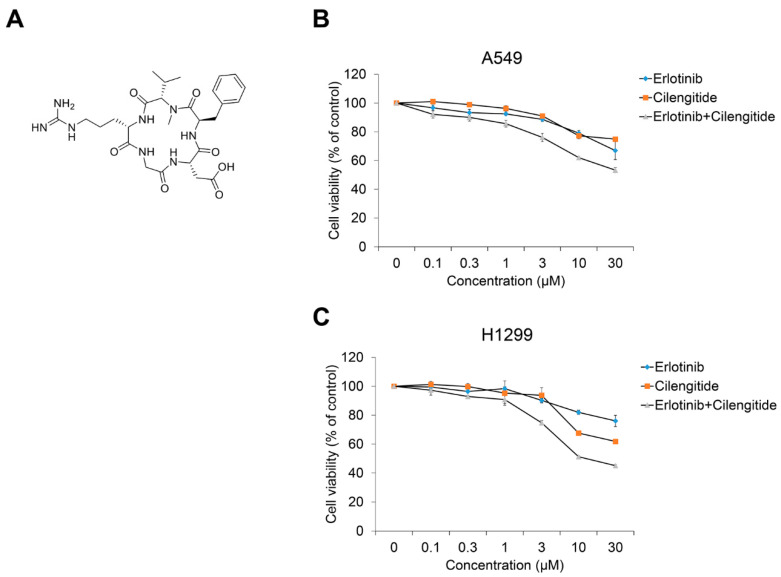
Cilengitide enhances the inhibitory effect of erlotinib on the cell viability of A549 and H1299 cells. (**A**) Chemical structure of cilengitide. (**B**) A549 and (**C**) H1299 cells were treated with erlotinib alone or with cilengitide for 24–72 h. After incubation, cell viability was measured by the CCK-8 assay. Experiments were performed in triplicate. Data represent mean ± SD.

In a previous report on cilengitide combined with the EGFR inhibitor gefitinib, we confirmed their cytotoxicity in CPAE cells and a synergistic effect on NSCLC cell viability and TGF-β1–induced EMT phenotype changes [36]. Based on that study, we investigated the combined treatment effect of cilengitide with erlotinib on NSCLC cell viability and TGF-β1–induced EMT marker expression. As shown in Figure 4, compared to treatment with erlotinib or cilengitide (Figure 4A) alone, combined treatment exhibited a synergistic effect on A549 (Figure 4B) and H1299 cell proliferation (Figure 4C). The CI calculated using the raw data confirmed this result (Table 1 and Table S2, Figure S8). Synergistic effects were observed at concentrations >0.1 μM.

**Figure 5:**
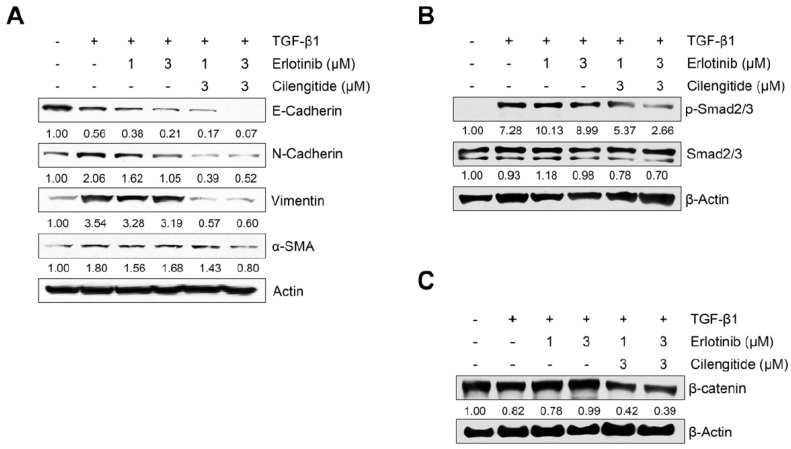
Effect of combined treatment on TGF-β1–induced EMT marker expression and Smad and non-Smad signaling. A549 cells were treated with gefitinib (1 μM) and cilengitide (3 μM) individually or in combination, and then incubated with TGF-β1 (5 ng/mL) for 72 h (**A**,**C**) or 48 h (**B**). (**A**) The expression of EMT markers was measured by Western blot analysis. Actin was used as a loading control. (**B**,**C**) The expression of protein was measured by Western blot analysis. β-actin was used as a loading control.

We also evaluated the combination effect of cilengitide with erlotinib on protein and mRNA expression of EMT markers in A549 cells. As shown in Figure 5A, although combination treatment did not lead to recovery of the TGF-β1–induced decrease in E-cadherin, inhibition of N-cadherin, vimentin, and α-SMA was stronger with combined treatment than with erlotinib alone. To confirm the synergistic effect of the two combined drugs, we investigated their influence on Smad and non-Smad signaling. As shown in Figure 5B, the combination showed stronger inhibition than either agent singly against TGF-β1–induced phosphorylation of Smad2/3. In addition, TGF-β1–induced expression of β-catenin was suppressed more with combined treatment (Figure 5C), although the combination did not affect phosphorylation of EGFR or ERK1/2 more than either agent alone (Figure S9). These results indicate that combined treatment may enhance the efficacy of erlotinib in inducing NSCLC cell death and inhibit the TGF-β1–induced EMT process through the suppression of Smad and non-Smad Wnt/β-catenin signaling.

### 2.4. The Combination of Cilengitide with Erlotinib Exerts a Synergistic Inhibitory Effect on Tgf-Β1–Mediated Invasion and MMP Secretion

**Figure 6:**
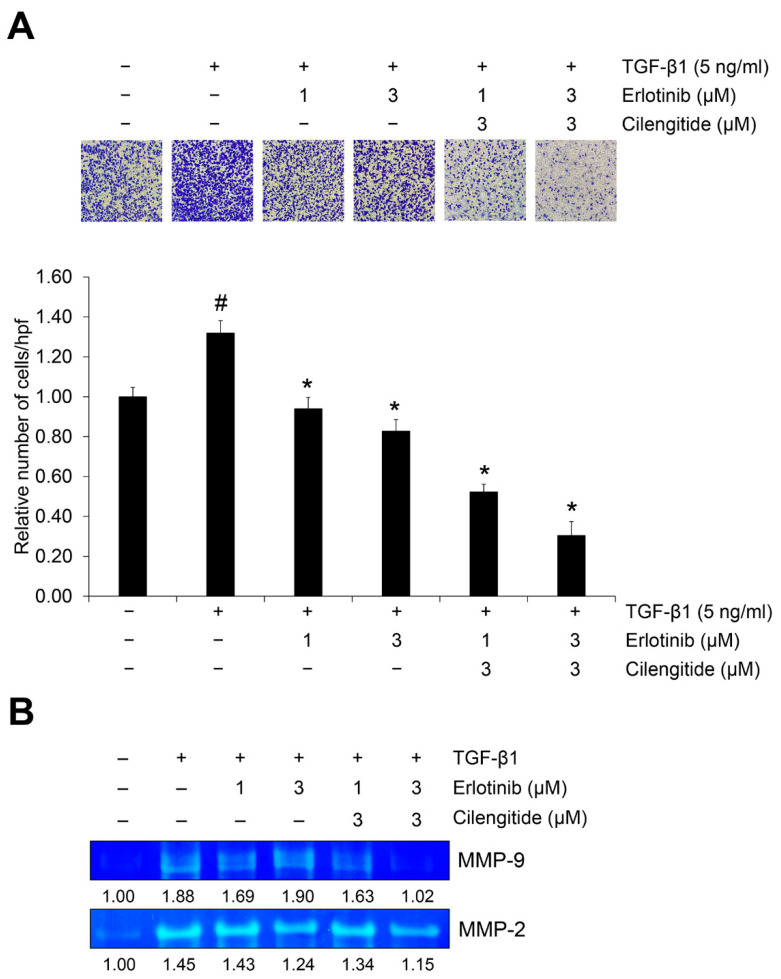
The combination of erlotinib and cilengitide exerts an enhanced inhibitory effect on TGF-β1–induced invasion and MMP activities in A549 cells. Serum-deprived A549 cells were treated with TGF-β1 (5 ng/mL) and erlotinib alone or in combination with cilengitide for 48 h. (**A**) The effect of the combination on TGF-β1–induced invasion of A549 cells was evaluated using Boyden chambers. Numbers of invaded cells were represented by an average number of cells per randomly selected three high-power field (HPF). (**B**) Activation of MMP-2 and MMP-9 was measured by gelatin zymography. ^#^ *p* < 0.01 versus control; * *p* < 0.05 versus the group treated with TGF-β1 only.

We also tested the effect of cilengitide with erlotinib on TGF-β1–induced invasion in A549 cells. In our previous report, we confirmed that cilengitide has an inhibitory effect and that combined treatment with gefitinib yielded a synergistic effect [36]. As shown in Figure 6A, either erlotinib or cilengitide inhibited the TGF-β1–induced invasion of A549 cells across the gelatin-coated membrane, and their combination increased this inhibitory effect.

**Figure 7:**
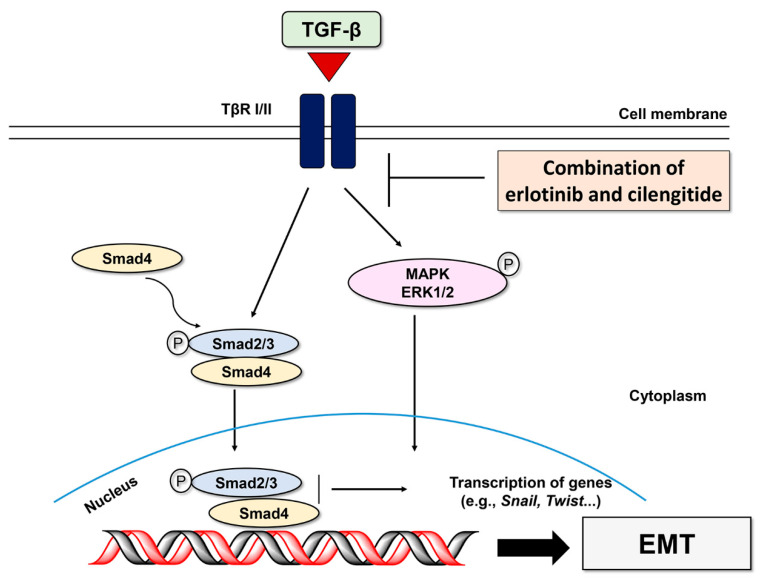
Schematic of TGF-β-mediated EMT signaling pathways inhibited by combined treatment of erlotinib and cilengitide.

During extracellular matrix (ECM) disruption, matrix metalloproteinases (MMPs) cleave the ECM in ways that promote shape changes in cancer cells so that they can maintain viability and develop invasive and migratory properties [37]. We used gelatin zymography to investigate the combination effect of the major gelatinases MMP-2 and MMP-9 on TGF-β1–induced EMT activation. As shown in Figures S10 and S11, erlotinib treatment inhibited TGF-β1–induced gelatinase activities of both MMP-2 and MMP-9, but cilengitide did not significantly affect them. However, the combination of erlotinib and cilengitide yielded stronger inhibition than either agent singly (Figure 6B). Collectively, these results indicate that combined treatment with erlotinib and cilengitide can exert enhanced inhibitory effects on the TGF-β1–induced invasion process (Figure 7).

## 3. Discussion

Here we show that co-administration of cilengitide with erlotinib increases the inhibitory effects of erlotinib alone on NSCLC cell survival and TGF-β1–induced EMT changes. Collectively, these results suggest that EFGR inhibitors such as gefitinib and erlotinib, when combined with cilengitide, may synergize in the treatment of EMT-related diseases, including human lung cancer, pulmonary fibrosis, and other metastatic cancers.

During the progression of lung cancer, cells acquire metastatic and invasive properties through the EMT process [3,38]. This process also is responsible for maintaining NSCLC cell survival by activating anti-apoptotic signals that both alter cell shape to a mesenchymal phenotype and confer resistance to chemotherapy [39]. Therefore, in the treatment of NSCLC, inhibition of EMT along with the induction of cancer cell death can be an important therapeutic strategy for suppressing cancer cell metastasis. We confirmed this hypothesis in the current study, showing that the cyclic pentapeptide cilengitide is effective as co-treatment with erlotinib, an EGFR-targeting drug that induces NSCLC cell death, to increase inhibitory efficacy against TGF-β1–induced EMT and reduce metastatic potential in NSCLC.

For the treatment of NSCLC, drugs such as the TKIs erlotinib and gefitinib are used in chemotherapy, but long-term administration may cause side effects, including cancer cell resistance and the death of healthy cells [39]. To reduce these side effects and increase therapeutic efficacy, studies have increasingly focused on combined treatment therapies such as immunotherapy, radiation therapy, and targeted therapy [22]. In general, with combination drug therapy for NSCLC treatment, side effects arising from the use of small molecule drugs can be reduced. In particular, cyclic peptides are being actively studied as biochemical tools and therapeutic agents because of their excellent in vivo stability and high affinity and selectivity for target molecule binding compared to linear peptides [40].

Cyclic peptides such as cRGDfK and cRGDyK exhibit high binding affinity and selectivity for the overexpressed integrins α_ν_β_3_ and α_ν_β_5_ in cancer cells, facilitating the delivery of anticancer drugs to the target [41,42]. We recently demonstrated that cRGDfK increased NSCLC apoptosis and enhanced the inhibitory effects of the TKI sunitinib on TGF-β1–induced EMT [43]. Single compound treatment with TKI involves toxic effects at high concentrations, but in that study, EMT could be effectively inhibited when a cyclic peptide was added to a low concentration of TKI in combined treatment. Combination therapy using cilengitide also has been suggested as a way to improve the therapeutic efficacy of cancer treatment [30,31,32,33,44]. Clinical trials to evaluate the enhancement co-administration effect of cilengitide with conventional drugs are also ongoing or have been conducted [29,33,34]. In the meantime, many clinical studies have been conducted on the effects of co-administration of erlotinib with other drugs [22,23,24,25]. However, another co-administration strategy is also required because there is a limit to the co-administration of small molecule drugs and the possibility for conversion into resistant cells. 

Although studies on cilengitide co-administration with various drugs are underway, the mechanism for EMT inhibition and the combination effects in NSCLC have not yet been examined. In a pair of studies on NSCLC cells, we have confirmed the inhibitory effect of cilengitide co-administration and the EGFR inhibitor gefitinib [36] or erlotinib (the current work) on EMT. Collectively, our findings show that the combination of cilengitide and EGFR inhibitors could represent a new chemotherapeutic option for modulating growth and invasive changes in EMT-associated NSCLC cell changes. Because EGFR inhibitors effectively inhibit downstream signaling even when used alone, we found little effect of cilengitide co-administration on EGFR signaling. The current study describes the co-administration effect of EGFR inhibitors and cilengitide only on EMT, and future studies should investigate the co-administration of cilengitide with other classes of NSCLC therapeutics. Among the various types of RGD-based cyclic peptides developed, cilengitide has been most investigated for its anticancer efficacy, but other peptides are understudied. In addition, with few studies evaluating the co-administration effect of small molecules and cyclic peptides in various diseases affected by EMT, the current findings offer a helpful basis for future research on inhibiting EMT-induced progression in other cancers. 

We are currently synthesizing cyclic RGD peptide derivatives such as cilengitide and studying the effects of NSCLC cell growth inhibition and EMT inhibition. Through a recent study and this study, we verified the efficacy of TKIs such as gefitinib and erlotinib and cyclic RGD peptides in EMT. Based on these results, we plan to continuously study the effect of a new cyclic RGD peptide on NSCLC treatment and increase anticancer effect through co-administration. In addition, despite these findings, there is a need to diversify the approach to dealing with EMT according to the characteristics of lung epithelial cells. For example, in squamous carcinoma, Notch and Wnt signaling are shown to be very important, whereas in adenocarcinoma, RAS mutations occur frequently and have different biology. Therefore, we need to address these issues in more depth in future studies.

## 4. Materials and Methods

### 4.1. Materials

Erlotinib was purchased from Sigma–Aldrich (St. Louis, MO, USA), and cilengitide was synthesized by Kyeong-Yong Park (CHA Meditech Co., Ltd., Daejeon, Korea) (Figures S1–S3). Fetal bovine serum (FBS), RPMI-1640 medium, and antibiotics (100 U/mL penicillin and 100 μg/mL streptomycin) were purchased from Corning Inc. (Corning, NY, USA). Recombinant human TGF-β1 was purchased from R&D Systems, Inc. (Minneapolis, MN, USA). Antibodies against E-cadherin (No. 3195), α-smooth muscle actin (α-SMA) (No. 19245), p-ERK1/2 (No. 4370), ERK1/2 (No. 4695), p-EGFR (No. 3777), EGFR (No. 4267), and p-Smad2/3 (No. 8828) were purchased from Cell Signaling Technology, Inc. (Danvers, MA, USA). Antibodies against Smad2/3 (No. sc-133098), vimentin (No. sc-6260), N-cadherin (No. sc-59987), and β-catenin (No. sc-7199), horseradish peroxidase (HRP)-conjugated secondary antibodies (No. sc-516102 and No. sc-2357), HRP-conjugated actin (No. sc-1615), and HRP-conjugated β-actin (No. sc-47778) were purchased from Santa Cruz Biotechnology, Inc. (Dallas, TX, USA).

### 4.2. Cell Culture and Cell Viability Assay

Bovine pulmonary epithelium CPAE (KCLB No. 10209), human lung fibroblast IMR90 (KCLB No. 10186), human NSCLC A549 (KCLB No. 10185), and H1299 (KCLB No. 91299) cell lines were obtained from Korean Cell Line Bank. All NSCLC and CPAE cells were maintained in RPMI-1640 medium (Corning, NY, USA), and IMR90 cells were maintained in minimum essential medium (Corning) containing 10% heat-inactivated FBS and 1% antibiotics (100 U/mL penicillin and 100 μg/mL streptomycin). All cells were maintained at 37 °C in a humidified atmosphere of 5% CO_2_.

To assess cell viability, cells were seeded in 96-well plates for 24 h. After incubation, erlotinib or cilengitide were added for 24–72 h in culture medium containing 10% FBS. Cell viability was assessed using EZ-Cytox Enhanced Cell Viability Assay Kit (DogenBio, Seoul, Korea) according to the manufacturer’s instructions. The absorbance was measured by a Multiscan^TM^ FC microplate photometer (Thermo Fisher Scientific, Waltham, MA, USA), and cell viability is presented as a percentage of control cells (untreated cells).

### 4.3. Western Blot Analysis

All cell lysates were prepared using Nuclear and Cytoplasmic Extraction Reagent (Thermo Fisher Scientific, Waltham, MA, USA). Quantified proteins were separated by SDS-PAGE, transferred to nitrocellulose membranes, and incubated with specific primary antibodies and HRP-conjugated secondary antibodies. We used all primary or secondary antibodies diluted in TBST buffer containing 5% skim milk at a ratio of 1:1000. The band intensities were visualized using SuperSignal^TM^ West Femto maximum sensitivity substrate (Thermo Fisher Scientific, Waltham, MA, USA) and measured using X-ray films and development solution (Fujifilm, Tokyo, Japan). The detected bands were quantified using ImageJ software (NIH, New York, NY, USA), and the relative ratios between each sample and the loading control are presented in this paper.

### 4.4. Invasion Assay

Serum-deprived A549 cells were treated with TGF-β1 (5 ng/mL) and erlotinib or cilengitide or their combination for 48 h. Cells were collected using trypsin-EDTA and resuspended in serum-free medium for counting. Culture medium (30 μL) containing 5% FBS was added to the bottom of a Boyden chamber. After placement of a gelatin-coated membrane filter, silicone gasket, and top chamber, the cell suspension (5 × 10^3^ cells/50 μL) was added to the top chamber and incubated at 37 °C in 5% CO_2_ for 4 h. The membrane filter was collected, fixed, and stained using the Diff-Quick Staining Kit (Thermo Fisher Scientific, Waltham, MA, USA) according to the manufacturer’s instructions.

### 4.5. Quantitative Real-Time PCR (qRT-PCR) Analysis

Total RNA was isolated using the AccuPrep^®^ RNA Extraction Kit (Bioneer Corp., Daejeon, Korea), and the cDNA was synthesized from 1 μg of total RNA using oligo (dT) primers (Bioneer Corp., Daejeon, Korea) and the RocketScript^TM^ Reverse Transcriptase Kit (Bioneer Corp., Korea). qRT-PCR was performed using Prime Q-Mastermix (GeNet Bio, Daejeon, Korea) and the CFX96^TM^ Real-Time System (Bio-Rad, Hercules, CA, USA). The cycling conditions were as follows: 95 °C for 3 min followed by 40 cycles at 95 °C for 15 s, 60 °C for 30 s, and 72 °C for 30 s. The primer sequences are shown in Table S1. All reactions were run in triplicate, and data were analyzed using the 2^−ΔΔCT^ method [41]. The internal standard was GAPDH. Significance was determined by the Student’s *t*-test with GAPDH-normalized 2^−ΔΔCT^ values [45].

### 4.6. Gelatin Zymography

A549 cells were incubated in a 6-well plate for 24 h. After serum starvation, cells were treated with TGF-β1 (5 ng/mL) and erlotinib or cilengitide, or their combination, for 48 h. The supernatants were collected, and centrifuged at 3000× *g* for 10 min, and protein was concentrated using Amicon^®^ Ultra Centrifugal Filters (Merck, Kenilworth, NJ, USA). After protein quantification using the BCA protein assay (Thermo Fisher Scientific, Waltham, MA, USA), proteins (40 μg) were loaded onto a gelatin-containing acrylamide gel and separated by electrophoresis. Protein separated gel was washed with 2.5% Tween-20 solution, developed overnight at 37 °C in Zymogram incubation buffer (50 mM Tris-HCl, pH 7.6; and 5 mM CaCl_2_), stained with 0.25% Coomassie blue R250 solution, and destained with a solution of 50% methanol and 10% acetic acid until the part of membrane degraded by MMP-2 or MMP-9 became clear. The activity of matrix metalloproteinase (MMP)-2 and MMP-9 was measured according to a previously described experimental procedure [46]. 

### 4.7. Analysis of Combined Drug Effects

We analyzed the effects of the drug combination using the CalcuSyn software program (Biosoft, Cambridge, UK). To determine whether the result of treatment with the two compounds was additive or synergistic, we applied the combination index (CI) derived from the median effect principle of Chou and Talalay (1984) [47]. The CI was calculated using the formula published by Zhao et al. (2004) [48]. A CI of 1 indicates an additive effect between the two compounds, a CI > 1 indicates antagonism, and a CI <1 indicates synergism.

### 4.8. Statistical Analysis

Data are presented as the mean ± SD of at least three independent experiments. Significant differences were evaluated by one-way ANOVA with post-hoc Tukey HSD test. *p* < 0.05 was considered significant.

## Figures and Tables

**Table 1:** Combination index (CI) values for the two-drug combination against A549 and H1299 cell viability.

Cell line.	Erlotinib (μM)	Cilengitide (μM)	CI Value
A549	0.1	0.1	0.2655
	0.3	0.3	0.5058
	1	1	0.6415
	3	3	0.2346
	10	10	0.0298
	30	30	0.0100
H1299	0.1	0.1	0.5450
	0.3	0.3	0.7446
	1	1	1.7137
	3	3	0.4097
	10	10	0.0357
	30	30	0.0359

## Data Availability

Data are contained within the article or supplementary material.

## Supplementary Materials

The following supporting information can be downloaded at: https://www.mdpi.com/article/10.3390/ijms23073423/s1.
