# Supplementary material for: Combination Effect of Cilengitide with Erlotinib on TGF-β1-Induced Epithelial-to-Mesenchymal Transition in Human Non-Small Cell Lung Cancer Cells
Source: Int J Mol Sci. 2022 Mar 22;23(7):3423. doi: 10.3390/ijms23073423 (PMC8999066; doi:10.3390/ijms23073423)
Supplement: Supplementary file 1 [file ijms-23-03423-s001.zip › ijms-1609436-supplementary.pdf]

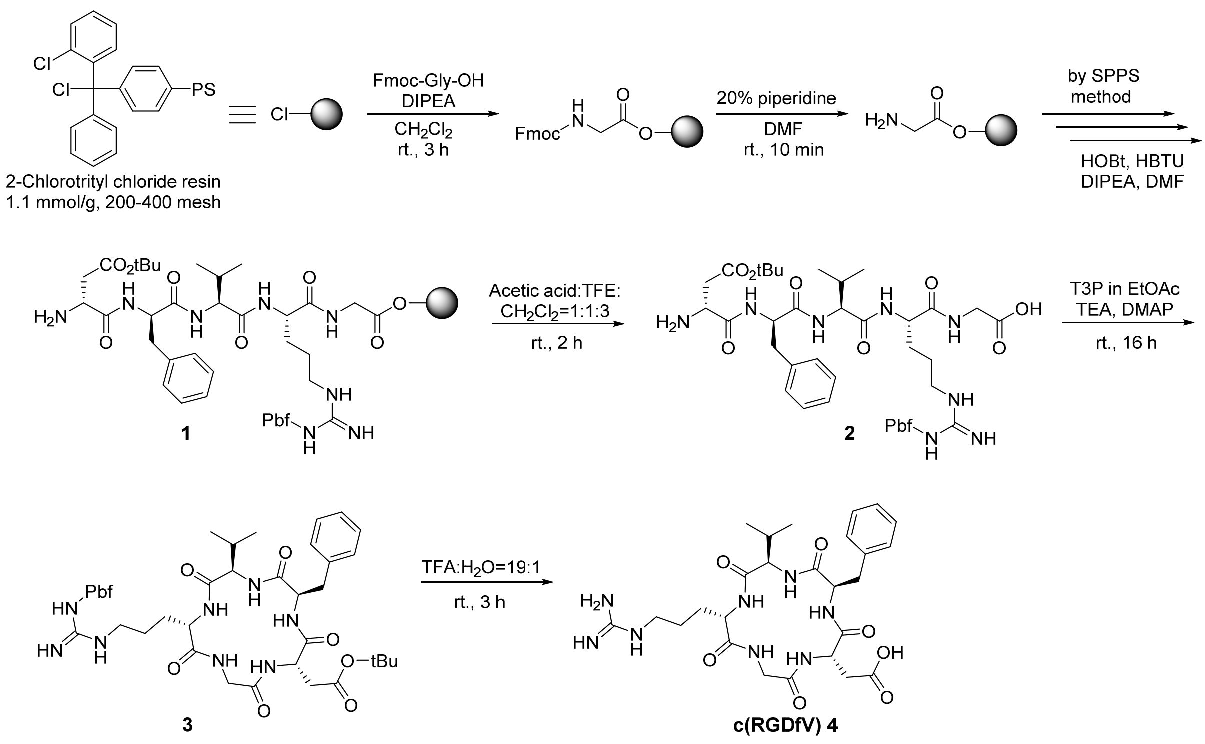


**Figure S1.** Synthetic scheme for cilengitide, c(RGDfV) **4**. Protected linear pentapeptide **1** bound to the resin was synthesised using the Fmoc solid-phase peptide synthesis (SPPS) method. Linear peptide **2** was cleaved from the resin by acetic acid/TFE/$CH_2Cl_2$ (1:1:3 ratio) solution without affecting other protecting groups. Finally, cyclic pentapeptide c(RGDfV) **4** was obtained by head-to-tail cyclisation under T3P, TEA, and DAMP, and elimination of the protecting group by TFA:$H_2O$=19:1(v/v).

# Experimental Section

## Mass spectrometry

Cilengitide masses were determined on a Waters ACQUITY UPLC H-Class/SQD2.

## HPLC spectrometry

HPLC analysis of cilengitide was conducted in $H_2O$ solution (1 mg/mL) using a Shimadzu HPLC 2030 model with a Vydac 218TP C18 column (5 µm, 4.6 × 250 mm). The A buffer was 0.1% trifluoroacetic acid (TFA) in $H_2O$, and the B buffer was 0.1% TFA in $CH_3CN$. The flow rate was 1 mL/min with a gradient of 0%–3% B in 3 min, 3%–60% B in 33 min, and 60% B in 35 min.

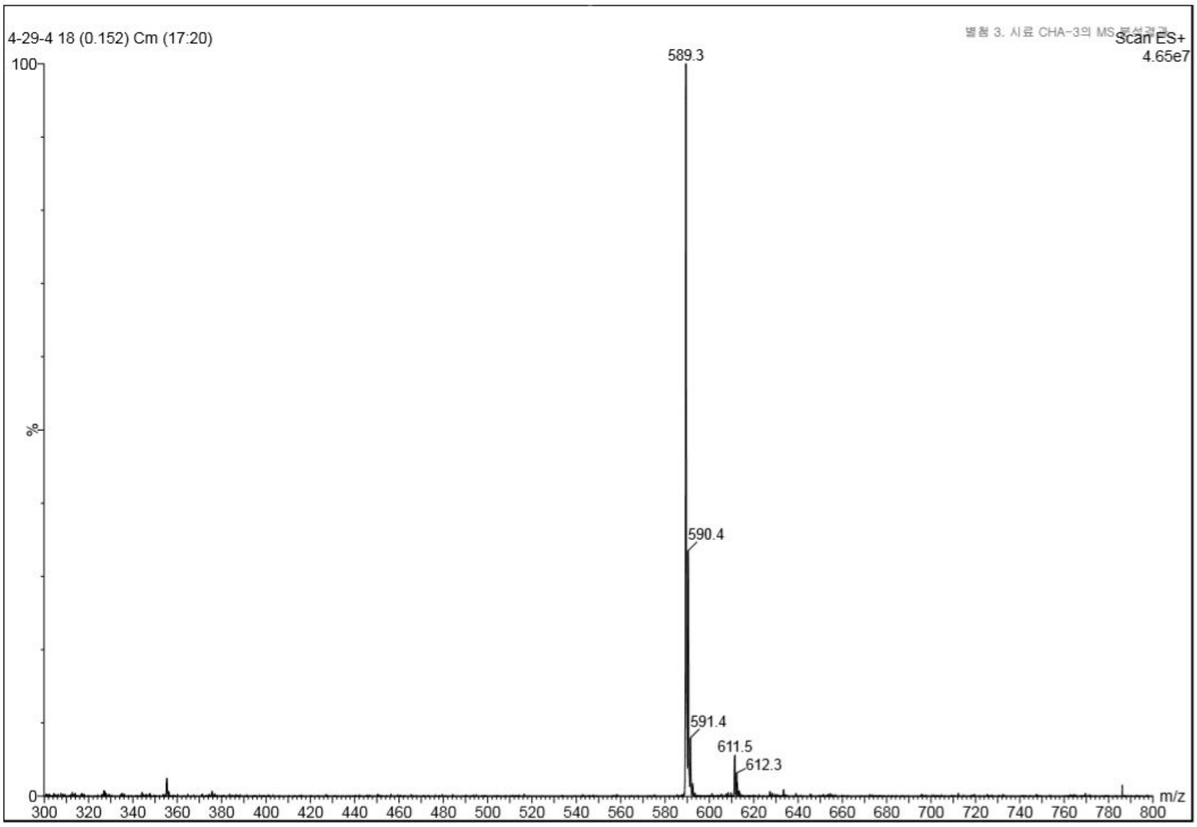


**Figure S2.** LC-mass spectrum of cilengitide. Molecular weight calculated, 588.6; observed, 589.3.

<Chromatogram>

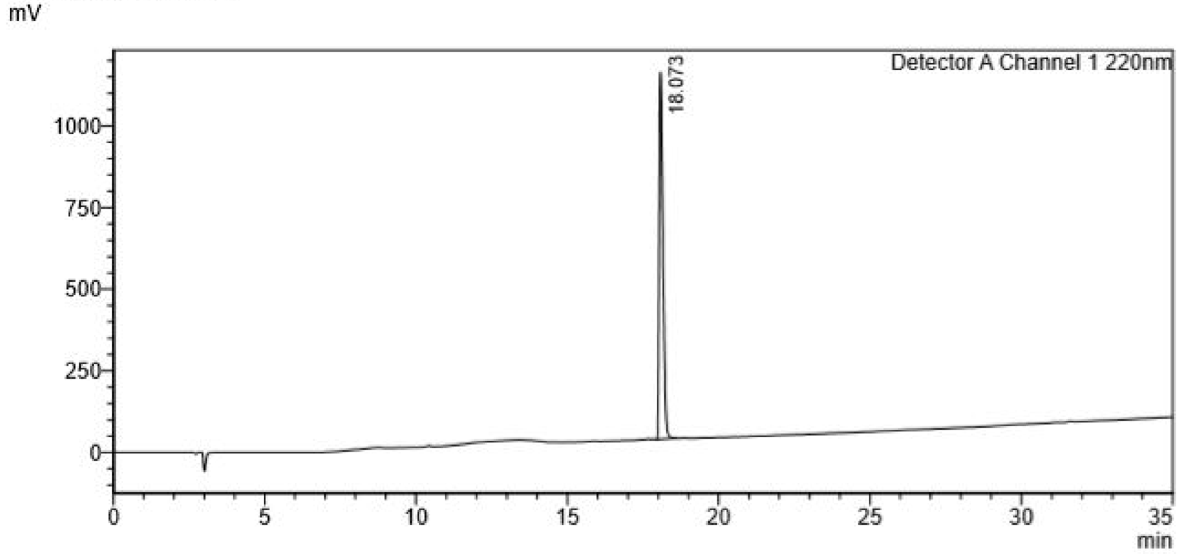


<Peak Table>

Detector A Channel 1 220nm

| Peak# | Ret. Time | Area | Area% | Height | Height% |
|---|---|---|---|---|---|
| 1 | 18.073 | 9840631 | 100.000 | 1123116 | 100.000 |
| Total | | 9840631 | 100.000 | 1123116 | 100.000 |

**Figure S3.** HPLC spectrum of cilengitide.

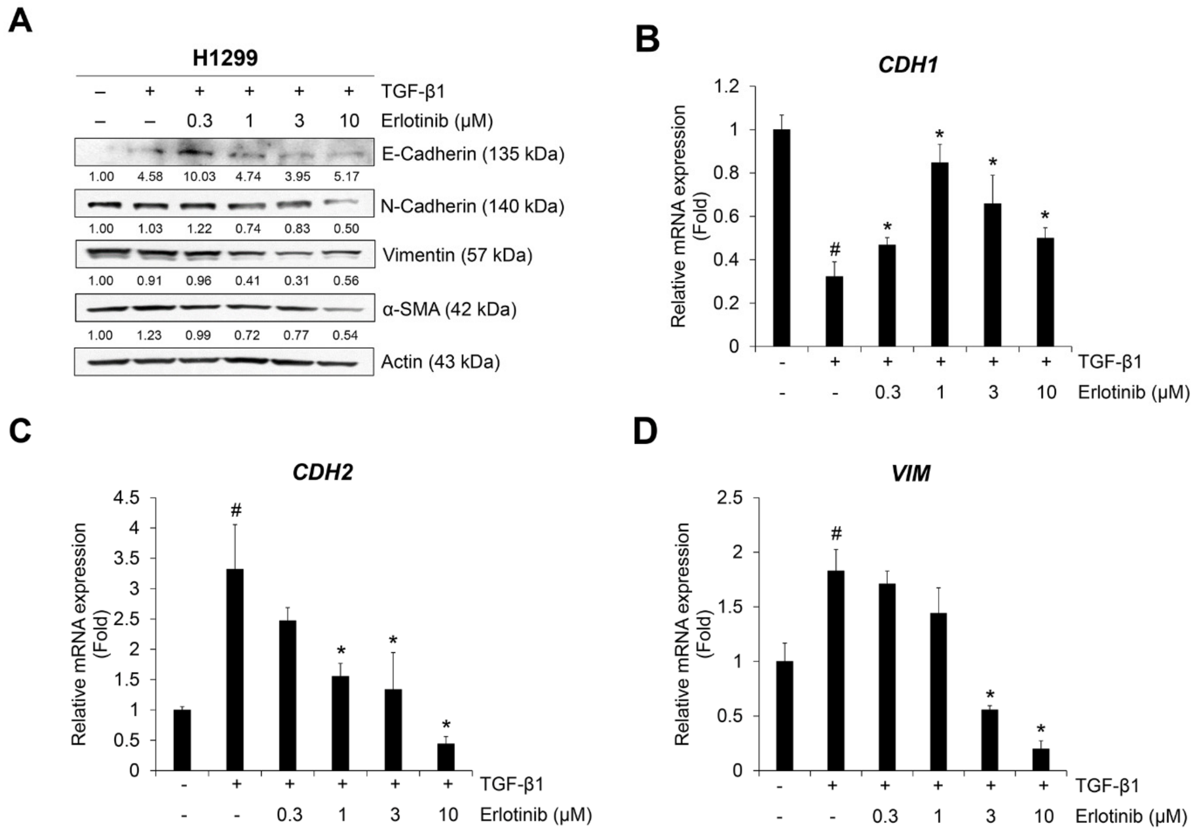


**Figure S4.** Erlotinib inhibits TGF-β1–induced expression of EMT markers in H1299 cells. (**A**) Serum-deprived H1299 cells were treated with TGF-β1 (5 ng/mL) or in combination with erlotinib for 72 h. Protein expression of an epithelial marker (E-cadherin) and mesenchymal markers (N-cadherin, vimentin, and α-smooth muscle actin) was determined by western blot analysis. Actin was used as a loading control. (**B**-**D**) Serum-deprived A549 cells were incubated with TGF-β1 (5 ng/mL) and erlotinib for 48 h. After RNA extraction and cDNA synthesis, we performed qRT-PCR to measure the expression of CDH1, CDH2, and VIM mRNA using GAPDH as an internal control. $^{\#}$ $p < 0.01$ versus control; * $p < 0.05$ versus the group treated with TGF-β1 only.

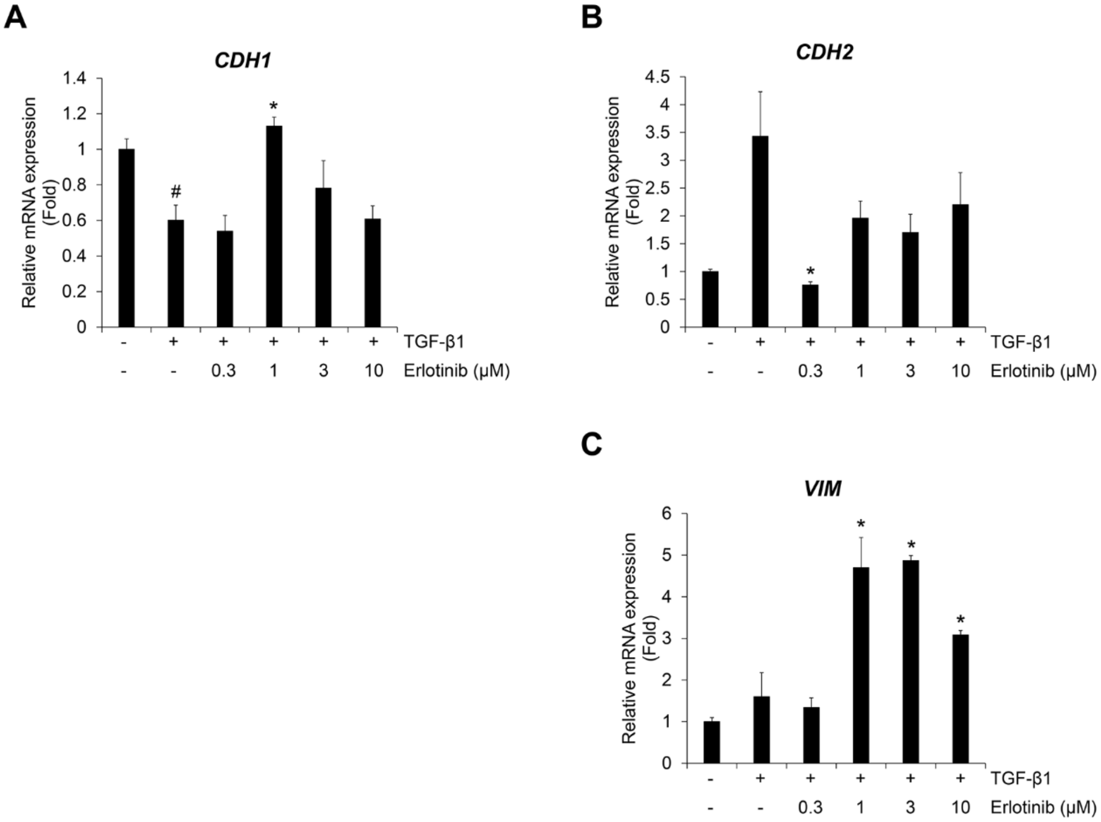


**Figure S5.** Effect of erlotinib on TGF-β1–induced expression of EMT marker genes in CPAE cells. (**A-C**) Serum-deprived A549 cells were incubated with TGF-β1 (5 ng/mL) and erlotinib for 48 h. After RNA extraction and cDNA synthesis, we performed qRT-PCR to measure the expression of CDH1, CDH2, and VIM mRNA using GAPDH as an internal control. $^{\#} p < 0.01$ versus control; * $p < 0.05$ versus the group treated with TGF-β1 only.

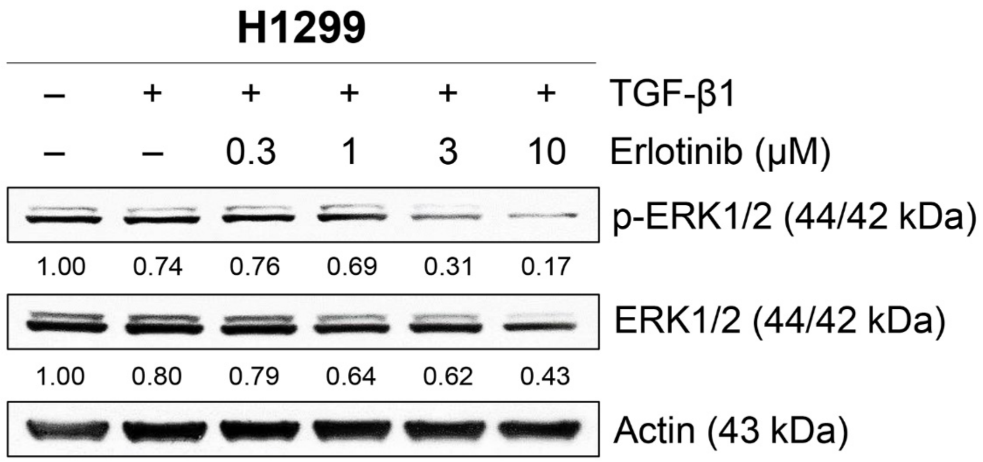


**Figure S6.** Erlotinib inhibits TGF-β1–induced non-Smad signalling in H1299 cells. Serum-deprived H1299 cells were treated with TGF-β1 (5 ng/mL) or in combination with erlotinib for 48 h. Phosphorylation of ERK1/2 was determined by western blot analysis. Actin was used as a loading control.

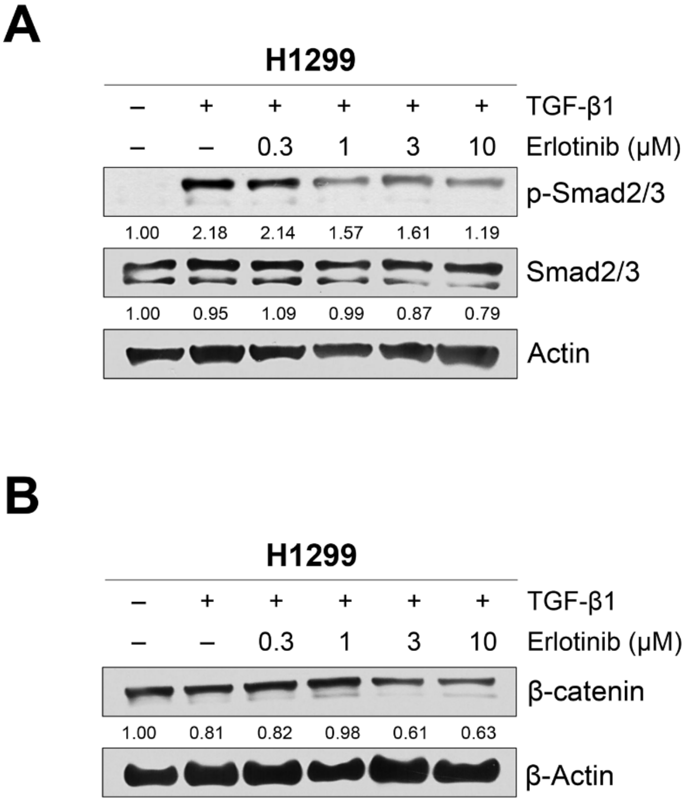


**Figure S7.** Erlotinib inhibits TGF-β1–induced Smad and non-Smad signalling in H1299 cells. Serum-deprived H1299 cells were treated with TGF-β1 (5 ng/mL) or in combination with erlotinib for 48 h. Phosphorylation of Smad2/3 (A) and expression of cytosolic β-catenin was determined by western blot analysis. Actin and β-actin were used as loading controls.

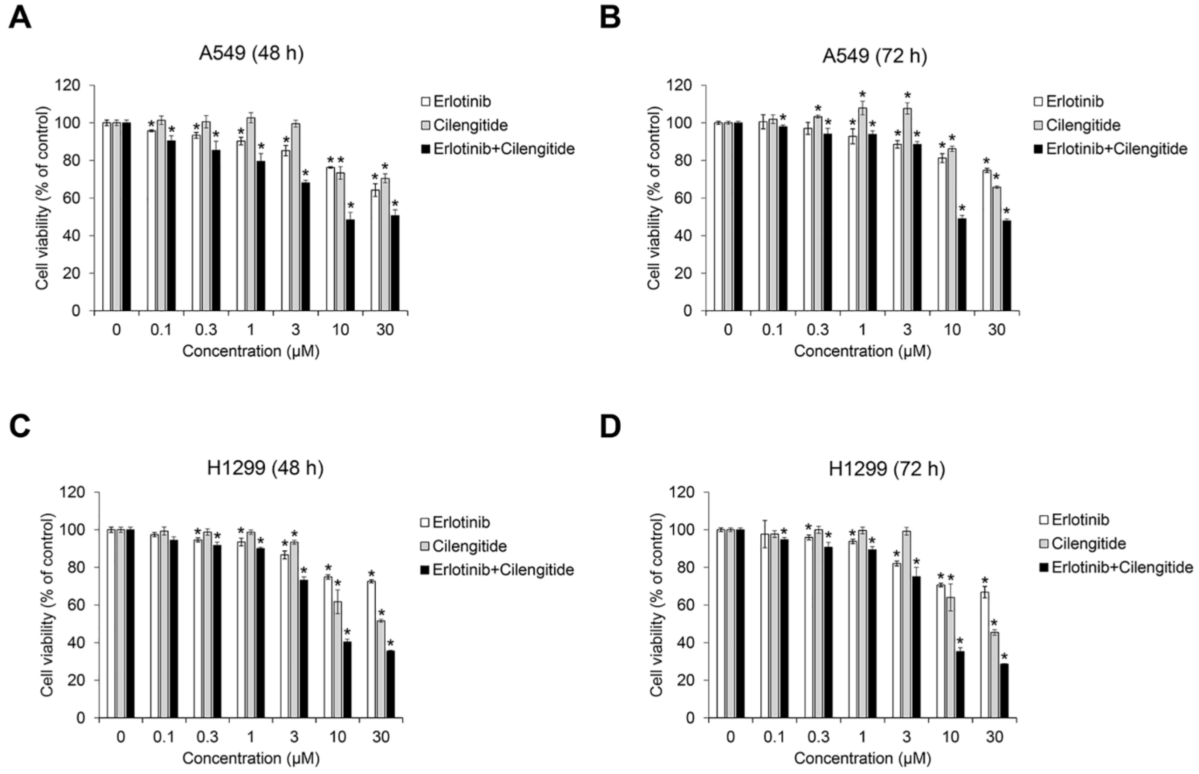


**Figure S8.** Cilengitide enhances the inhibitory effect of erlotinib on the viability of A549 and H1299 cells. (**A** and **B**) A549 and (**C and D**) H1299 cells were treated with erlotinib alone or in combination with cilengitide for 48 or 72 h. After incubation, cell viability was measured using the CCK-8 assay. Experiments were performed in triplicate. Data represent mean ± SD. * $p < 0.05$ versus untreated control.

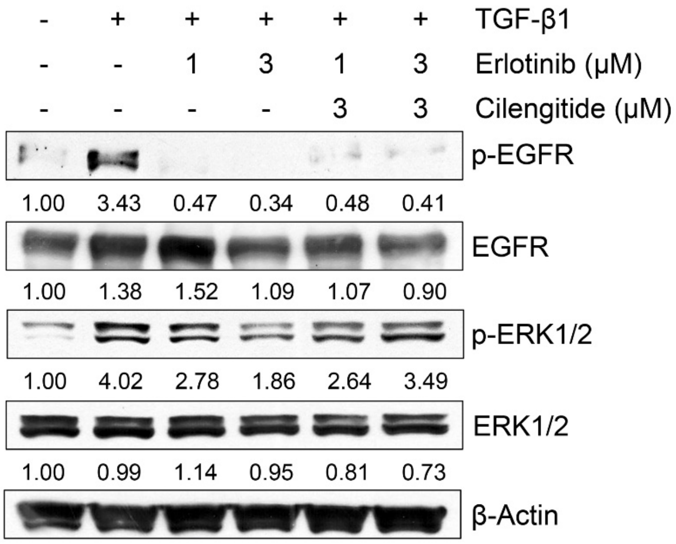


**Figure S9.** Effect of combined treatment on TGF-β1–induced non-Smad signalling. A549 cells were treated with gefitinib (1 μM) and cilengitide (3 μM) individually or in combination, and then incubated with TGF-β1 (5 ng/mL) for 72 h. Protein expression was measured by western blot analysis. Actin was used as a loading control.

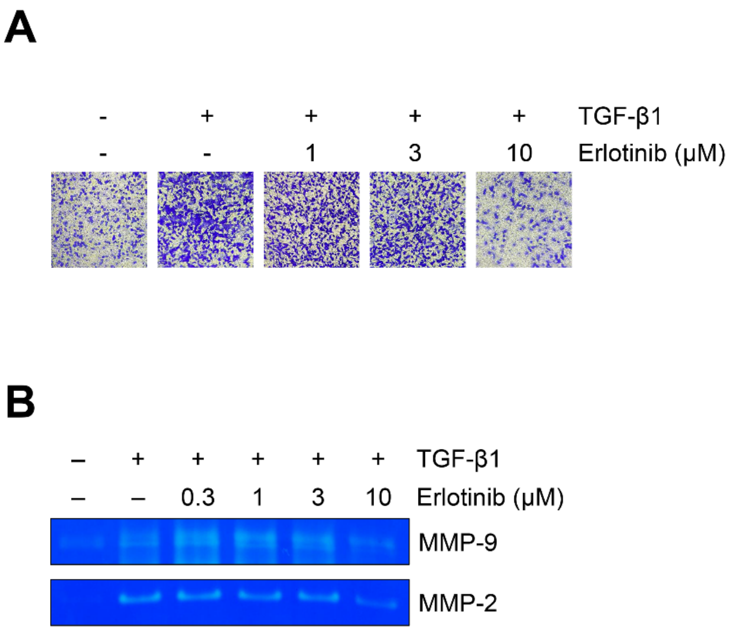


**Figure S10.** The effect of erlotinib on TGF-β1–induced invasion and MMP activity in A549 cells. Serum-deprived A549 cells were treated with TGF-β1 (5 ng/mL) and erlotinib for 48 h. (**A**) The effect of erlotinib on TGF-β1–induced invasion of A549 cells was evaluated using Boyden chambers. (**B**) Activation of MMP-2 and MMP-9 was measured by gelatine zymography.

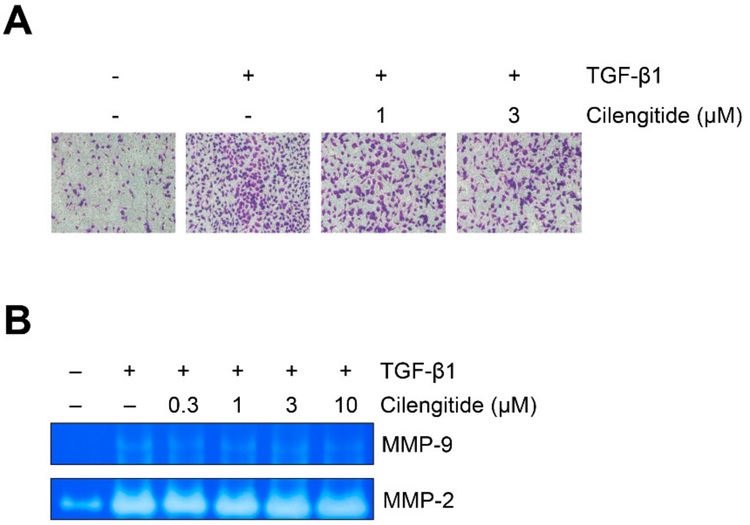


**Figure S11.** The effect of cilengitide on TGF-β1–induced invasion and MMP activity in A549 cells. Serum-deprived A549 cells were treated with TGF-β1 (5 ng/mL) and cilengitide for 48 h. (**A**) The effect of cilengitide on TGF-β1–induced invasion of A549 cells was evaluated using Boyden chambers. (**B**) Activation of MMP-2 and MMP-9 was measured by gelatine zymography.

**Table S1.** Sequences of primers used in this study.

| Target gene | Forward (5' – 3') | Reverse (5' – 3') |
|---|---|---|
| *CDH1* | TCCGAAGCTGCTAGTCTGAG | CTCAAGGGAAGGGAGCTGAA |
| *CDH2* | CCCACAGCTCCACCATATGA | TTCAGTCATCACCTCCACCA |
| *VIM* | CGCCAACTACATCGACAAGG | GGCTTTGTCGTTGGTTAGCT |
| *GAPDH* | GAGTCAACGGATTTGGTCGT | GATCTCGCTCCTGGAAGATG |

**Table S2.** Combination index (CI) values for the two-drug combination against A549 cell viability.

| Cell line | Incubation time (h) | Erlotinib (μM) | Cilengitide (μM) | CI value |
|---|---|---|---|---|
| A549 | 48 h | 0.1 | 0.1 | 0.2425 |
| | | 0.3 | 0.3 | 0.3032 |
| | | 1 | 1 | 0.3484 |
| | | 3 | 3 | 0.1171 |
| | | 10 | 10 | 0.0049 |
| | | 30 | 30 | 0.0256 |
| | 72 h | 0.1 | 0.1 | 0.4949 |
| | | 0.3 | 0.3 | 0.6539 |
| | | 1 | 1 | 2.0795 |
| | | 3 | 3 | 2.0498 |
| | | 10 | 10 | 0.0015 |
| | | 30 | 30 | 0.0034 |
| H1299 | 48 h | 0.1 | 0.1 | 0.4995 |
| | | 0.3 | 0.3 | 0.9604 |
| | | 1 | 1 | 2.4855 |
| | | 3 | 3 | 0.7146 |
| | | 10 | 10 | 0.0247 |
| | | 30 | 30 | 0.0314 |
| | 72 h | 0.1 | 0.1 | 0.4168 |
| | | 0.3 | 0.3 | 0.5463 |
| | | 1 | 1 | 1.4124 |
| | | 3 | 3 | 0.3775 |
| | | 10 | 10 | 0.0004 |
| | | 30 | 30 | 0.0002 |
